# Liver and Bilateral Tubo-Ovarian Abscesses Caused by Streptococcus constellatus in an Immunocompetent Adolescent: A Case Report

**DOI:** 10.7759/cureus.68319

**Published:** 2024-08-31

**Authors:** Marien Govea, Enrique Konstat-Korzenny, Pablo Laufer

**Affiliations:** 1 Pediatrics, Florida International University, Herbert Wertheim College of Medicine, Miami, USA; 2 Pediatrics, Nicklaus Children's Hospital, Miami, USA; 3 Pediatrics/Infectious Disease, Nicklaus Children's Hospital, Miami, USA

**Keywords:** streptococcus constellatus, abscess, hepatic abscess, immunocompetent, infectious disease, adolescent, pediatric

## Abstract

Opportunistic infections most often occur in immunocompromised patients, however, they can also occur in immunocompetent patients. While rare, bacterial infections such as those from *Streptococcus constellatus* (*S. constellatus*) can cause severe pyogenic infections and abscess formations. It is important to understand the risk factors, diagnostic workup, and management of patients with this rare but enduring bacterial infection. Although most of the literature reports the prevalence of *S. constellatus* in adults, occurrences should not be overlooked in the pediatric population. We present a case of an immunocompetent 19-year-old female who initially presented with refractory fevers and was found to have an intrahepatic abscess and bilateral tubo-ovarian abscesses. Management of this patient included percutaneous drainage of the hepatic abscess and antibiotic treatment for 28 days.

## Introduction

Liver abscesses are usually polymicrobial, with the most common bacterial infections caused by *Escherichia coli, Klebsiella, and Streptococcus.* *Streptococcus constellatus (S. constellatus)* is a rare bacterium that colonizes the oral, colonic, and urogenital mucosa [1]. It is a gram-positive streptococcus that can cause severe pyogenic infections in the regions it colonizes or hematogenously spreads to.

Most reports on *S. constellatus* infections involve adult patients. These are mainly pyogenic and there are several reports of liver, brain, lung, and abscesses in other parts of the body [2]. We herein present a pediatric case of a previously healthy adolescent female who presented with refractory fevers and was found to have liver and bilateral tubo-ovarian abscesses secondary to *Streptococcus constellatus*. The in-patient diagnosis and treatment are detailed so that physicians are aware of this uncommon cause of pyogenic liver abscesses. The intent of this report is to bring awareness to this bacteria since its presentation in the pediatric population is uncommon.

## Case presentation

A 19-year-old female presented initially to the emergency department (ED) complaining of two days of fever and one day of pelvic pain. Maximum axillary temperature taken was 103 F. She denied any other associated factors. She visited her OB/GYN the prior week where physical exam and urinalysis were within normal limits. Workup in the ED revealed urinalysis with positive leukocyte esterase, nitrites, and bacteria. Pregnancy test was negative. Abdominal X-ray with findings compatible with fecal retention but otherwise unremarkable. She tested positive for influenza B and was discharged with oseltamivir treatment to continue as an outpatient for five days.

One week later, the patient returned to the ED as her fever persisted despite oseltamivir and now presented a two-day course of sharp right flank pain, rated 7/10, worsened with movement. At the time she denied nausea, vomiting or diarrhea. Upon further questioning, her mother endorsed a past medical history of recurrent *E. coli *urinary tract infections, resolved vesicoureteral reflux (which on further chart review was diagnosed as Grade 2) and occasional albuterol use while having upper respiratory infection symptoms in the past. She denied any significant family history of neoplasms. Patient also denied history of recurrent infections or immunodeficiencies. She denied any alcohol, tobacco, or drug usage. She lived in a household with her mother, had not traveled outside of the USA but had traveled recently to the state of Colorado. There were no pets at home. She was sexually active with one male partner with consistent barrier contraception use but had a previous male partner with inconsistent barrier contraception methods. Last menstrual period reported one week prior to ED visit.

An abdominal and pelvic ultrasounds were ordered. The pelvic report showed complex bilateral adnexal collections, the right ovary measured 5.3 x 4.6 x 3.5 cm, calculated volume of 45.4 mL. The left ovary measured 7.4 x 5.3 x 5.0 cm, calculated volume of 102.6 mL.” These findings were concerning for pylosalpinx. 

The abdominal ultrasound showed enlarged kidneys (2 SD above for age) with preserved anatomy and a heterogeneous mass in the right liver lobe with internal flow upon Doppler interrogation measuring approximately 10.0 x 7.6 x 9.3 cm. Also found to have hepatomegaly with a longitudinal span of 21 cm. Basic workup revealed leukocytosis, elevated liver transaminases, and elevated C-reactive protein. Urinalysis was similar to the one observed on the initial visit.

**Figure 1:**
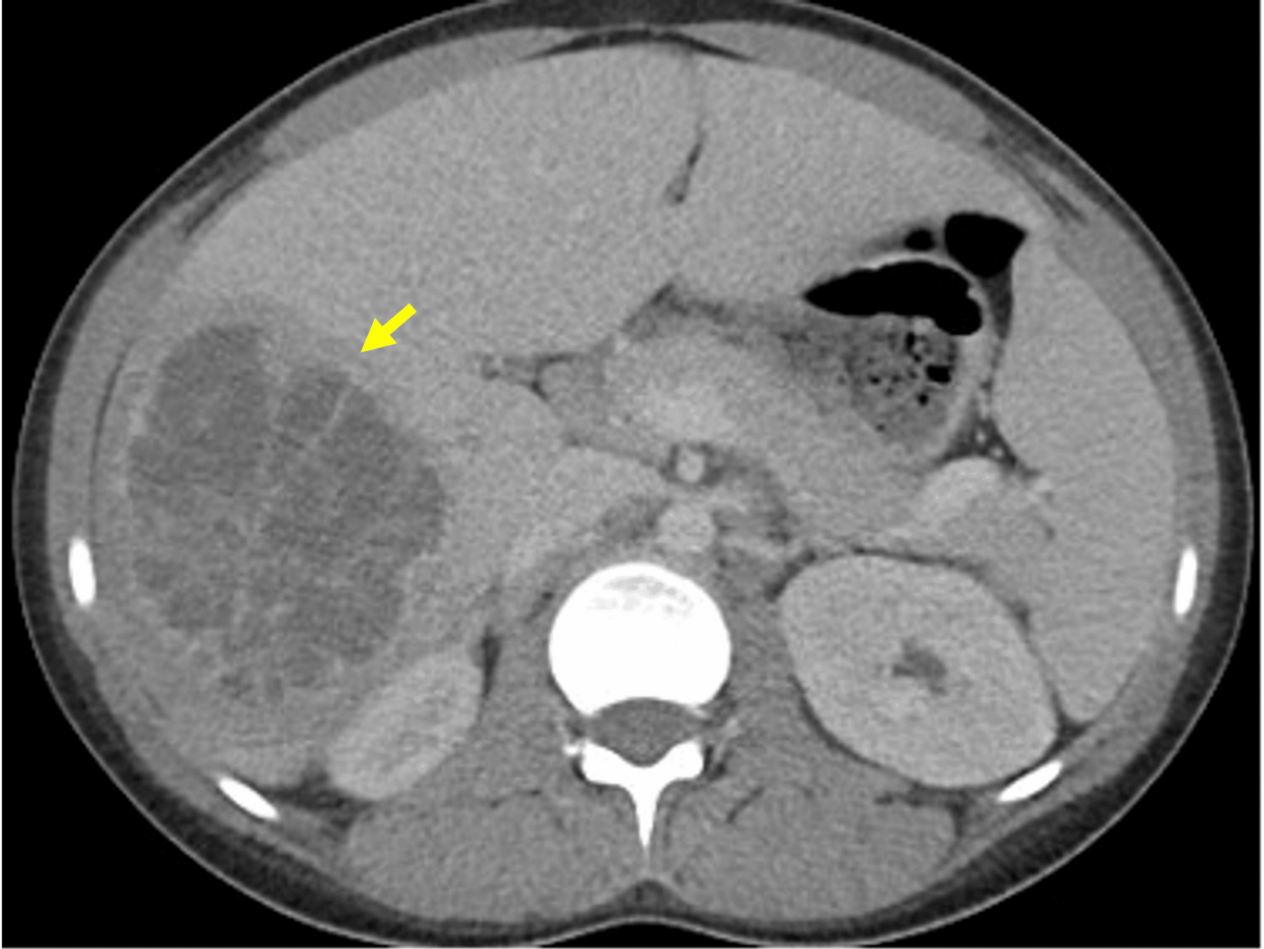
Abdominal CT with findings of right hepatic mass with surrounding edema

Out of concerns for pelvic inflammatory disease (PID) as bilateral pylosalpinx was observed, she was started on standard treatment with ceftriaxone, doxycycline, and metronidazole. Blood culture was obtained and remained negative at 48 hours; urine culture turned positive at 24 hours for pansensitive *E. coli*. At the time *Chlamydia *and *Gonorrhea* polymerase chain reaction (PCR) were obtained and returned negative at 24 hours. HIV, hepatitis B virus (HBV) and rapid plasma reagin (RPR) tests were negative. CT of the abdomen showed a heterogeneous, multiseptated right hepatic mass with surrounding hepatic and periportal edema suggestive of an intrahepatic abscess, as well as enlarged, serpiginous and peripherally enhancing bilateral adnexa suggestive of pyosalpinx and bilateral tubo-ovarian abscesses with reactive free fluid (Figure 1).

She was admitted for further workup of the hepatic imaging representing a likely abscess. A vaginal and pelvic exam was done which demonstrated negative cervical motion tenderness exam. Cervical swabs were taken for *Gonorrhea* and *Chlamydia* and were negative five days later. Thus, PID was excluded from the differential.

**Figure 2:**
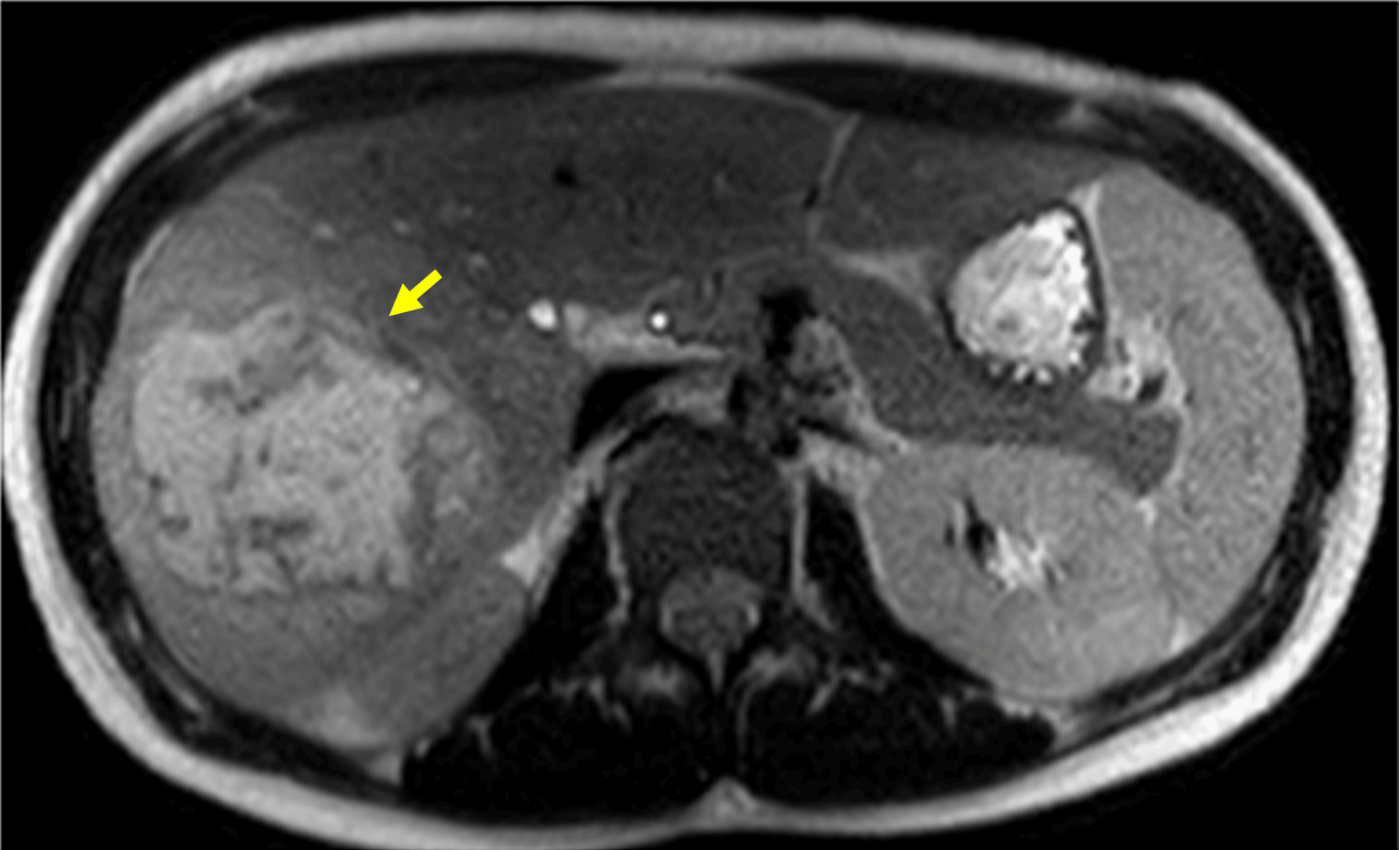
Abdominal MRI with findings of a large, complex and septated collection in the right lobe of the liver

An MRI to further characterize the hepatic lesion reported the liver measuring 23 cm in the craniocaudal dimension. A large lobulated complex collection with multiple septations measuring 10.5 x 8.1 x 10.1 cm was found in the right lobe. Also reported bilateral tubo-ovarian abscesses with small amount of pelvic free fluid (Figure 2).

Infectious disease service recommended continuing with the current treatment. The differential diagnosis included a pyogenic abscess, a hydatid cyst or amebic abscess, although the latter was less likely as no epidemiological risk factors were observed. Her clinical picture did not improve despite antibiotic treatment. She persisted with abdominal pain and fevers throughout the day, especially at night. She remained distended and with hepatomegaly. On day five of admission, interventional radiology performed a percutaneous drainage of the lesion and a total of 310 cc of frank pus were removed, collapsing the cavity. The pus became blood-tinged at the end. Acid-fast bacilli (AFB), fungal, aerobic, and anaerobic cultures were obtained. A post-drainage ultrasound reported an interval decrease in the size of the previously seen complex abscess measuring approximately 9.2 x 8.8 cm. The hemoglobin dropped to 7.3 g/dL so she was started on oral iron supplementation.

She improved significantly after drainage of the abscess. Her fever curve decreased and she no longer presented fevers at night. Her abdominal pain decreased significantly although it was still present especially while moving. She was able to eat and ambulate as usual.

Since* Gonorrhea* and *Chlamydia* cultures were negative and the gram stain reporting Gram-positive organisms, her antibiotic treatment was switched to piperacillin/tazobactam monotherapy. *Echinococcus* and *Entamoeba* panel was negative. Acid-fast bacteria and fungal cultures remained negative. A stool calprotectin sample was obtained and resulted negative. Diphtheria, Haemophilus influenzae, and tetanus titers were within normal limits. Immunodeficiency was essentially ruled out with a panel that resulted normal. Culture of the obtained pus was positive for *S. constellatus*, a member of the *S. anginosus* family. A sensitivity panel returned and showed the bacterium was sensitive to cephalosporins. She was then switched back to ceftriaxone monotherapy on day 10 and a peripherally inserted central catheter (PICC) line was placed to continue with treatment for three to four weeks at home with close follow-up with infectious disease service.

One week later repeat ultrasounds reported reduction of the right ovary size, measured at 3.6 x 3.3 x 2.0 cm with a calculated volume of 12.6 mL (previously 45 mL), and the left ovary measured 6.0 x 4.7 x 3.3 cm with a calculated volume of 49.4 mL (previously 103 mL). Regarding the hepatic abscess, the lesion measured approximately 5.4 x 6.3 x 5.4 cm. Previously the lesion was measured at 9.6 x 8.8 x 8.3 cm. The liver remained enlarged measuring 19.1 cm in length. Her overall inflammatory markers improved and clinically she reported improvement in her abdominal pain. The patient returned for follow-up after 23 days of ceftriaxone via PICC line. At the time of follow-up she endorsed an erythematous rash that responded to antihistamine treatment therefore she was switched to oral, high-dose amoxicillin. On day 28 of antibiotics, she continued with the dermatological reaction. At that time, labs showed a decrease in both WBC and inflammatory markers therefore antibiotic therapy was discontinued.

On her follow-up visit two months after discharge, she reported feeling well overall. Repeat ultrasound of the ovaries showed a right ovary that measured 3.3 x 2.9 x 2.4 cm, a calculated volume of 12.2 mL. The left ovary measured 4.4 x 2.4 x 2.4 cm, a calculated volume of 13.4 mL, thus improving from previous study. Liver ultrasound showed a decrease in size of the known right hepatic lobe lesion, measuring 2.4 x 1.8 x 2.3 cm versus 4.6 x 4.5 x 3.3 cm on prior exam. No new lesions were identified. Right longitudinal liver span is 15.0 cm, which is the upper limit of normal. Last follow-up appointment about four months after intervention proved no evidence of the hepatic mass and no distortion in overall architecture of the liver.

## Discussion

There is little evidence of Streptococcus constellatus infection in the immunocompetent pediatric population.

The Streptococcus anginosus group are opportunistic pathogens that can lead to severe pyogenic infections. They are found in the oral, digestive, pulmonary, and urinary tract. In a case report published in 2021 by Vulisha et al., a 59-year-old male presented with a cavitary lung lesion formed by Streptococcus constellatus that was complicated by empyema. The abscess formation in this patient was secondary to a lower extremity deep vein thrombosis and pulmonary embolism. The patient was treated with 35 days of ertapenem [3]. Similarly, in 2021 Xia et al. reported a 71-year-old man with an empyema infection that improved with piperacillin-tazobactam. This patient also presented with refractory fevers and associated cough and chest pain for which workup included next-generation sequencing of the fluid drained on a right-sided pleural effusion [2]. Other case reports demonstrate that this pathogen can also cause cardiac tamponade in immunocompetent adolescents, suppurative thrombophlebitis, hepatic abscesses among others [4-6]. The most common presenting symptom in these patients is the refractory fevers that initiate an extensive workup. Another case report published in 2020 by Mora-Palma et al. describes a 55-year-old female who was found to have cervical discharge positive for S. constellatus after the removal of a seven-year-old intrauterine device [7]. While sexually active, the adolescent patient discussed in this case did not have any intrauterine device that would increase her risk for such an infection. Thus, the bilateral tubo-ovarian abscesses were considered to be of consequence to the liver abscess. The majority of the cases mentioned above allude to the hematogenous dissemination of Streptococcus infection, which may help explain the multiple abscesses found in the adolescent patient. 

Risk factors for opportunistic infections such as this one include being an elderly male, smoking history, alcohol abuse, neoplastic disease, diabetes mellitus, chronic respiratory disorders, hematologic disorders, heart failure, HIV, trauma, surgical procedures, and even periodontal disease [8]. None of these risk factors applied to this adolescent patient at the time of diagnosis. 

Liver abscesses are predominantly classified as either bacterial or amoebic. The majority are reported to be polymicrobial with E. coli and K. pneumoniae as the most commonly isolated bacteria. Septic emboli can lead to abscesses and various reports reveal hematogenous spread from endocarditis, pyelonephritis, and diverticulitis sources. Liver abscesses from amoebic sources are commonly due to Entamoeba histolytica which often seeds into the portal system. An even rarer form of liver abscesses can stem from parasitic Echinococcus granulosus causing a hydatid cyst [1]. However, our case adds to two other reports that indicate that Streptococcus constellatus is a pathogen found in the formation of liver abscesses. One case published in 2021 by Riaz et al. discusses a 41-year-old male patient with a history of diverticulitis who presented with multiple liver abscesses and was consequently discharged with three weeks of levofloxacin and metronidazole via a PICC line [9]. In 2016, Akuzawa et al. also reported a 69-year-old male patient with multiple liver abscesses that progressed to bacteremia caused by Streptococcus constellatus [10]. This report emphasized the importance of timely diagnosis to prevent more severe consequences of this infection. 

Failing to diagnose and appropriately treat pyogenic liver abscesses can be fatal. Right upper quadrant ultrasound can be a useful tool to visualize hepatic lesions, but it fails to accurately detect small focal lesions. Abdominal CT scan provides a more accurate view of hepatic lesions in various phases that may demonstrate the rim-enhancement or cavitary lesions in better light. The use of both modalities increases diagnostic capabilities [7].

Ultrasound and CT can also be used to drain the abscess. In small abscesses that are less than 5 mm, simple needle aspiration may be sufficient. Abscesses larger than 5 mm may require catheter placement for drainage of pus. Surgery is particularly helpful in cases of peritonitis. Finally, cultures and sensitivities will aid in determining the best course of management [1].

There are several treatment strategies when it comes to pyogenic liver abscesses. Empiric treatment begins with broad-spectrum antibiotics, usually third-generation cephalosporins and metronidazole. In a report by Riaz et al., an adult patient was found to have two complex liver abscesses of S. constellatus and was treated with two weeks of parenteral antibiotics through PICC line [8]. In another two case reports, oral piperacillin and tazobactam for two weeks helped achieve successful recovery in patients with abscess formations of S. constellatus. Other common antibiotics include cephalosporins and penicillins [11,12].

## Conclusions

The clinical presentation of abdominal pain and refractory fevers observed in this patient can include a variable list of etiologies. While delving deeper into the physical exam and the various studies performed to determine the cause of her hepatomegaly, it is important to keep bacterial abscess formation in the differential. Although a healthy, immunocompetent adolescent, the drainage of the abscess yielded a rare form of Streptococcus that was sensitive to cephalosporins. Treatment with ceftriaxone for 23 days via the PICC line and oral amoxicillin thereafter helped achieve improvement of symptoms at the two-month follow-up. Although an unusual etiology, *Streptococcus constellatus* has been reported to cause severe pyogenic infection in several organ systems, predominantly the lungs and the liver. The pediatric population may not have the same risk factors for such bacterial infection when compared to a more immunocompromised adult. Treatment is begun with broad-spectrum antibiotics and later corrected based on the results of sensitivity panels in both adults and children. This case demonstrates the diagnostic workup of an adolescent who was found to have a hepatic abscess, one of the common presentations of this opportunistic bacteria. Immunocompetence is not a protective factor for such infections and high vigilance should be placed on antibiotic treatments with proven sensitivities to prevent fatal complications. 
